# Ten-year outcomes of endothelial keratoplasty versus penetrating keratoplasty in Hong Kong: a retrospective cohort study

**DOI:** 10.1186/s12886-026-05332-4

**Published:** 2026-09-11

**Authors:** Anson Chun Long Wu, Ka Wai Kam, Eugenie Mok, Stephanie Hiu Wai Kwok, Alvin Lerrmann Young

**Affiliations:** 1 Department of Ophthalmology and Visual Sciences, Prince of Wales Hospital, Shatin, New Territories, Hong Kong SAR China; 2 Department of Ophthalmology and Visual Sciences, Alice Ho Miu Ling Nethersole Hospital, Tai Po, New Territories, Hong Kong SAR China; 3 Department of Ophthalmology and Visual Sciences, The Chinese University of Hong Kong, Shatin, New Territories, Hong Kong SAR China

**Keywords:** Keratoplasty, Endothelial keratoplasty, Penetrating keratoplasty, Fuchs endothelial dystrophy, Pseudophakic bullous keratopathy.

## Abstract

**Background:**

This study compares the long-term survival of primary endothelial (DSEK/DSAEK) versus penetrating keratoplasty in Hong Kong patients with Fuchs endothelial dystrophy (FED) or pseudophakic bullous keratopathy (PBK).

**Methods:**

A ten-year retrospective cohort of patients who received primary Descemet stripping (automated) endothelial keratoplasty (DSEK/DSAEK) or penetrating keratoplasty (PKP) for either FED or PBK at an academic ophthalmology department in Hong Kong between 2006 and 2015 was conducted.

**Results:**

Seventy-four eyes of 65 patients received primary keratoplasties with 95 months of mean follow-up, comprising 41 DSEK/DSAEK and 33 PKP. Overall graft survival rates were 76.1% at 5 years and 55.0% at 10 years. DSEK/DSAEK demonstrated higher survival rates than PKP, with 82.1% versus 68.8% survival at 5 years (*P* = 0.31) and 63.8% versus 43.5% at 10 years (*P* = 0.15). Older age was associated with graft failure risk. DSEK/DSAEK eyes had better observed best-corrected visual acuity (BCVA) at 5 years (*P* = 0.006), although this difference was attenuated after adjustment for preoperative BCVA, and the visual difference was no longer apparent at 10 years (*P* = 0.42). Graft ulcers occurred in fewer eyes receiving DSEK/DSAEK (4.9%) compared to PKP (21.2%) at 10 years (*P* = 0.033). The cumulative rejection rates were 12.2% for DSEK/DSAEK and 9.1% for PKP at 10 years (*P* = 0.67) while 14.6% of DSEK/DSAEK and 12.1% of PKP required a re-graft (*P* = 0.75).

**Conclusions:**

Both primary DSEK/DSAEK and PKP provided meaningful long-term graft survival in this Hong Kong cohort of patients with FED or PBK. DSEK/DSAEK eyes had better observed BCVA at 5 years, although this association was attenuated after adjustment for preoperative BCVA, and the visual difference was no longer apparent at 10 years. DSEK/DSAEK was associated with fewer graft ulcers, while rejection and re-graft rates were similar between groups. These findings should be interpreted in the context of the retrospective design, baseline differences between treatment groups, small subgroup sample sizes, and the historical DSEK/DSAEK techniques represented in this cohort.

**Supplementary Information:**

The online version contains supplementary material available at https://doi.org/10.1186/s12886-026-05332-4.

## Background

Keratoplasty is the definitive treatment for eyes with irreversible corneal oedema secondary to the loss of corneal endothelium. Penetrating keratoplasty (PKP) is the classical full-thickness corneal transplant whereas endothelial keratoplasty (EK) replaces the dysfunctional endothelium with donor posterior lamellar tissue [1]. In recent decades, EK has risen in dominance because of smaller wounds, faster visual recovery, less surgically-induced astigmatism and often superior visual outcomes compared to PKP. However, from a technical perspective, EK carries a steep learning curve especially in Asian eyes with narrow anterior chambers [1–3]. Excessive manipulation of the endothelial graft may increase cell loss and lead to early graft failure. A meta-analysis of comparative studies of Descemet stripping automated endothelial keratoplasty (DSAEK) versus PKP with follow-up periods up to 5 years showed no significant difference in pooled graft survival rates, but superior improvement in best-corrected visual acuity (BCVA) for DSAEK patients [4]. Another recent systematic review comparing long-term outcomes of DSEK/DSAEK and PKP suggested a trend towards better 5-year graft survival after DSEK/DSAEK [5], but both studies revealed a lack of data at 10 years. In 2020, we published our own outcome at 5 years following Descemet stripping endothelial keratoplasties (DSEK) or PKP in patients with endothelial dysfunction and found comparable survival rates between both techniques [6]. The current study aims to explore graft survival and outcomes at a longer term.

## Methods

### Subject recruitment

This is a retrospective cohort study on patients who received primary DSEK/DSAEK or PKP for either FED or PBK at an academic ophthalmology department in Hong Kong between 2006 and 2015. We excluded patients who received deep lamellar endothelial keratoplasty (DLEK), patients aged younger than 8 years old, and patients with less than 3 months of postoperative follow-up.

### Surgical technique

For DSEK/DSAEK, the donor disc was prepared manually on an artificial anterior chamber using lamellar dissectors for DSEK or pre-cut with automated microkeratome by the Eye Bank for DSAEK. A 4.5–5.0 mm scleral tunnel wound was created 1.5–2.0 mm behind the temporal limbus. If cataract extraction was required, this could be done either by phacoemulsification or extracapsular extraction techniques. The Descemet’s membrane was stripped under viscoelastic cover and a 7.5–8.5 mm donor endothelial lenticule was inserted as a 60/40 taco or with a sheet glide in the anterior chamber. The anterior chamber was filled with 12% SF6 for tamponade for 10 min then the gas was partially released to the size of donor graft. Topical homatropine 2% eye drop was applied to achieve a sustained pupil dilatation. Postoperatively, patients were instructed to lie strictly supine for at least two hours and usually for the first night after operation. Intraocular pressure was checked at one hour, three hours and the next morning. Patient was instructed to stay supine for most of the time, allowing brief upright position for meals or toilet. For PKP, an 8.0–8.5 mm donor punch was used and a suction trephine of 0.5 mm smaller diameter (i.e. 7.5–8.0 mm) was applied to the recipient. The host cornea was removed and the cataract was delivered during open-sky after either a continuous curvilinear capsulorhexis or a can-opening capsulotomy. The donor button was then secured with 16 interrupted 10/O nylon sutures after reforming iridocorneal angles and applying copious amount of viscoelastic in the wound bed. Both groups of patients were prescribed with topical prednisolone 1% eyedrops every 2 h during daytime and levofloxacin 0.5% eyedrops four times a day for the first week. Steroid eyedrops were tapered to four times a day over three months and once daily by 6 months postoperatively. They were kept on once daily topical prednisolone 1% indefinitely.

### Data collection

A standardised data collection sheet was used to collect demographic data including sex, age and ethnicity, preoperative data including diagnosis, waiting time for a corneal graft, preoperative BCVA, intraocular pressure (IOP) measured by non-contact tonometry, supplemented by Goldmann or iCare tonometry when the IOP was greater than 21 mmHg, central corneal thickness (CCT) measured by pachymetry, medical or ocular comorbidities such as hypertension, diabetes, hyperlipidaemia, ischaemic heart disease, autoimmune disease and glaucoma, and intraoperative data including type of surgery, operative time and intraoperative complications in order to compare baseline characteristics. Snellen visual acuities were converted to logMAR for computation. The following values were assigned to vision of counting fingers (1.4), hand movement (2.7), light perception (3.7) and no light perception (4.7) [7].

### Outcomes

The primary outcomes were graft survival rates of DSEK/DSAEK and PKP at 5 and 10 years. We also compared secondary outcomes including primary graft failure, re-graft rate, postoperative BCVA, IOP, CCT and complications such as graft rejection, graft ulcer, acutely raised IOP, chronically raised IOP, glaucoma, retinal or choroidal detachment, endophthalmitis, peripheral anterior synechiae, graft displacement and suture-related complications. The definition of graft failure was based on the definition used in the Collaborative Corneal Transplantation Studies [8]. Graft failure was defined by the occurrence of a re-graft for any reason, or a cloudy cornea with loss of central graft clarity sufficient to compromise vision for at least 3 consecutive months. The definition of graft rejection was consistent with the Cornea Donor Study, which included the presence of an endothelial rejection line or inflammation (keratic precipitates, cells in the stroma, or an increase in aqueous cells from a previous visit, with or without clinically apparent change in recipient stromal thickness or clarity) [9], treated with at least 2-hourly topical steroids or systemic steroids or cyclosporine. Acutely raised IOP was defined as IOP greater than 21 mmHg within 2 weeks postoperatively including transient postoperative IOP spikes, as confirmed by Goldmann applanation. Chronically raised IOP was defined as IOP greater than 21 mmHg at any timepoint postoperatively requiring initiation or addition of glaucoma medications for at least 3 consecutive months, while glaucoma was defined by an increased cup-disc ratio greater than 0.5, or optical coherence tomography of the retinal nerve fibre layer showing thinning.

### Statistical analysis

Descriptive data was reported with mean and standard deviation where appropriate. Chi-squared tests and student’s t-tests were applied to test for differences in categorical and continuous variables between groups respectively. Cumulative probabilities of graft survival were calculated using the Kaplan-Meier method and analysed with log rank test. Missing or lost follow-up data or patients who passed away during the study were censored. Univariate Cox regression analysis was used to assess factors associated with graft survival. Significant variables in univariate analysis were incorporated into multivariate analysis. Linear regression analysis was performed to identify factors associated with postoperative BCVA. Statistical analyses were conducted using R, and a *P* value of < 0.05 was considered statistically significant. DSEK and DSAEK were grouped together and analysed collectively since their surgical techniques were similar. Subgroup analysis of these two procedures was not performed due to the small sample size.

## Results

Seventy-four eyes of 65 patients received primary keratoplasties for either FED or PBK between 2006 and 2015, comprising 41 EK (35 DSEK and 6 DSAEK) and 33 PKP (Table 1). The mean duration of follow-up was 95 months (range: 3 to 224 months).

### Demographic and preoperative evaluation

Patients receiving DSEK/DSAEK and PKP were similar in age and sex (*P* > 0.05, Table 1). All except for 2 patients in the DSEK/DSAEK group were Chinese. There were more FED patients in the DSEK/DSAEK group (58.5% vs. 41.5%, *P* = 0.00014). Preoperative BCVA was better in the DSEK/DSAEK group (logMAR 1.08 ± 0.53 vs. 1.84 ± 0.73, *P* = 0.000029). Preoperative IOP and CCT were similar (both *P* > 0.05).

### Intraoperative procedure

Duration of surgery was significantly longer for DSEK/DSAEK as manual dissection of the corneal graft was mostly required (102.9 ± 26.1 vs. 88.8 ± 26.8 min, *P* = 0.042), and DSEK/DSAEK was more often combined with cataract extraction (53.7% vs. 15.2%, *P* = 0.00063, Table 1). An intraocular lens (IOL) was implanted in all patients who received simultaneous cataract extraction, while 7 IOLs required scleral fixation at the time of PKP following removal of the original anterior chamber IOL. Intraoperatively, DSEK/DSAEK was complicated by hyphaema in 3 patients, while there were 2 cases of IOL expulsion (including 1 case with vitreous expulsion), 1 case of vitreous prolapse, 1 broken scleral-fixated IOL suture and 1 DSEK conversion to PKP in the PKP group.

**Table 1:** Demographics, preoperative and intraoperative features

	All (*n* = 74)	DSEK/DSAEK (*n* = 41)	PKP (*n* = 33)	*P* values
Sex				
Male	29 (39.2%)	15 (36.6%)	14 (42.4%)	0.61
Female	45 (60.8%)	26 (63.4%)	19 (57.6%)	
Age at surgery (years)	74.7 ± 9.0 (range: 44–89)	73.6 ± 9.10 (range: 44–89)	76.1 ± 8.64 (range: 49–87)	0.25
Ethnicity				
Chinese	72 (97.3%)	39 (95.1%)	33 (100.0%)	0.20
Non-Chinese	2 (2.7%)	2 (4.9%)	0 (0.0%)	
Laterality				
Right	38 (51.4%)	19 (46.3%)	19 (57.6%)	0.34
Left	36 (48.6%)	22 (53.7%)	14 (42.4%)	
Diagnosis				
FED	29 (39.2%)	24 (58.5%)	5 (15.2%)	0.00014*
PBK	45 (60.8%)	17 (41.5%)	28 (84.8%)	
Preoperative BCVA (logMAR)	1.41 ± 0.73	1.08 ± 0.53	1.84 ± 0.73	0.000029*
Preoperative IOP (mmHg)	12.7 ± 3.51	12.3 ± 3.68	13.4 ± 3.10	0.29
Preoperative CCT (µm)	634 ± 54.8	630 ± 57.9	655 ± 23.0	0.46
Comorbidities				
Hypertension	63 (85.1%)	36 (87.8%)	27 (81.8%)	0.47
Diabetes	33 (44.6%)	16 (39.0%)	17 (51.5%)	0.28
Hyperlipidaemia	35 (47.3%)	25 (61.0%)	10 (30.3%)	0.0086*
Ischaemic heart disease	11 (14.9%)	7 (17.1%)	4 (12.1%)	0.55
Autoimmune disease	6 (8.1%)	2 (4.9%)	4 (12.1%)	0.26
Glaucoma	14 (18.9%)	5 (12.2%)	9 (27.3%)	0.10
Duration from listing to surgery (days)	621 ± 477	496 ± 365	803 ± 556	0.018*
Duration of surgery (minutes)	96.7 ± 27.3	102.9 ± 26.1	88.8 ± 26.8	0.042*
Combined surgery				
With cataract extraction	27 (36.5%)	22 (53.7%)	5 (15.2%)	0.00063*
Type of cataract extraction				
ECCE	1 (1.4%)	0 (0.0%)	1 (3.0%)	0.041*
Phacoemulsification	18 (24.3%)	14 (34.1%)	4 (12.1%)	
SLIMCE	8 (10.8%)	8 (19.5%)	0 (0.0%)	
With IOL implantation	34 (45.9%)	22 (53.7%)	12 (36.4%)	0.14
Position of IOL				
IOL in the bag	27 (36.5%)	22 (53.7%)	5 (15.2%)	0.000058*
SFIOL	7 (9.5%)	0 (0.0%)	7 (21.2%)	
Intraoperative complications	8 (10.8%)	3 (7.3%)	5 (15.2%)	0.28
Mean duration of follow-up (months)	94.8 ± 53.6	100.2 ± 55.3	88.2 ± 50.6	0.34

BCVA: best-correct visual acuity; CCT: central corneal thickness; DSEK/DSAEK: Descemet stripping (automated) endothelial keratoplasty; ECCE: extracapsular cataract extraction; FED: Fuchs endothelial dystrophy; IOL: intraocular lens; IOP: intraocular pressure; PBK: pseudophakic bullous keratopathy; PKP: penetrating keratoplasty; SFIOL: scleral-fixated intraocular lens; SLIMCE: sutureless large-incision manual cataract extraction

*P < 0.05

### Graft survival at 5 and 10 years

**Fig. 1:**
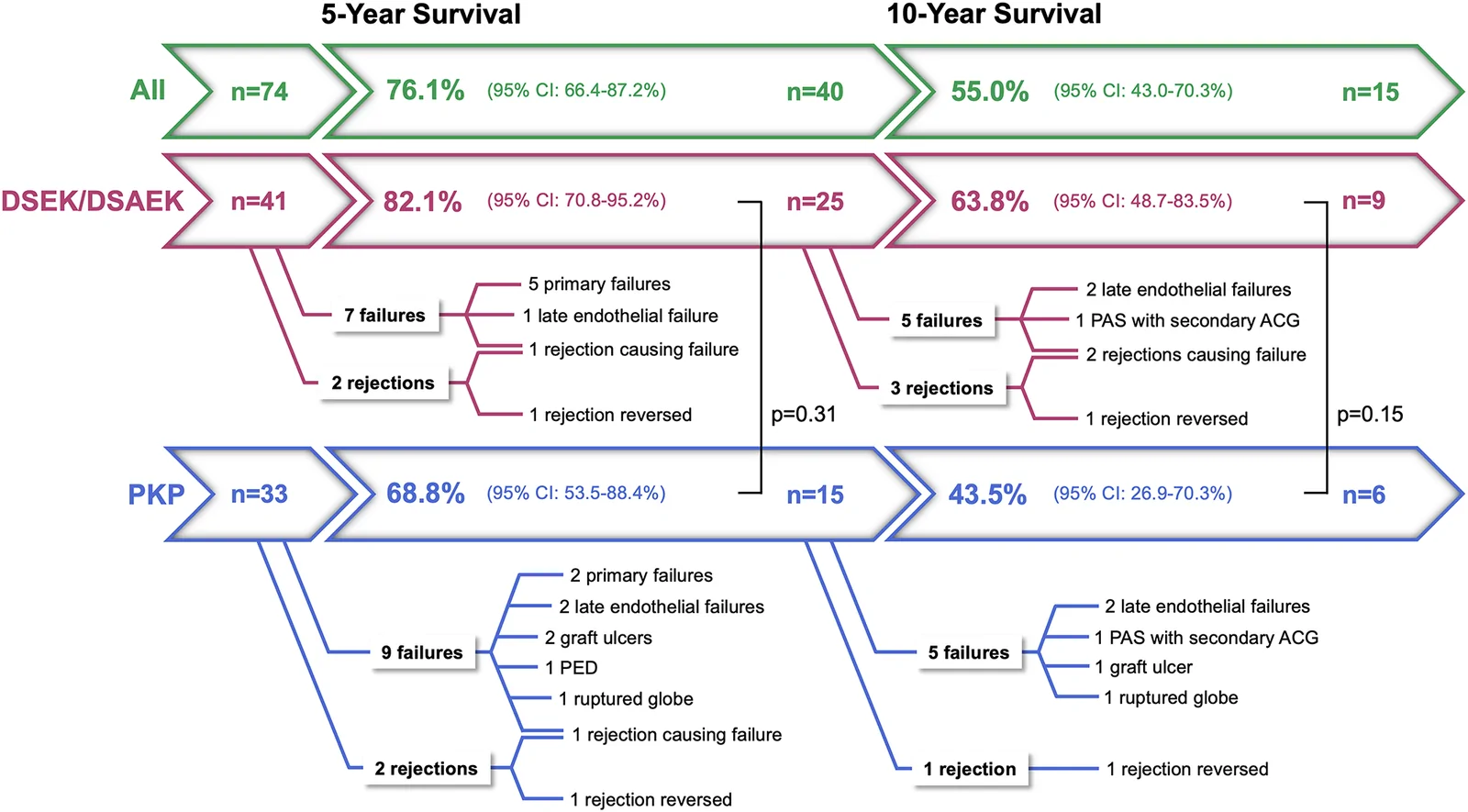
Graft survival and outcomes. Figure showing 5-year and 10-year survival rates for DSEK/DSAEK and PKP, causes of graft failure and outcomes of graft rejections. ACG: angle closure glaucoma; CI: confidence interval; DSEK/DSAEK: Descemet stripping (automated) endothelial keratoplasty; n: number at risk; PAS: peripheral anterior synechiae; PED: persistent epithelial defect; PKP: penetrating keratoplasty

**Fig. 2:**
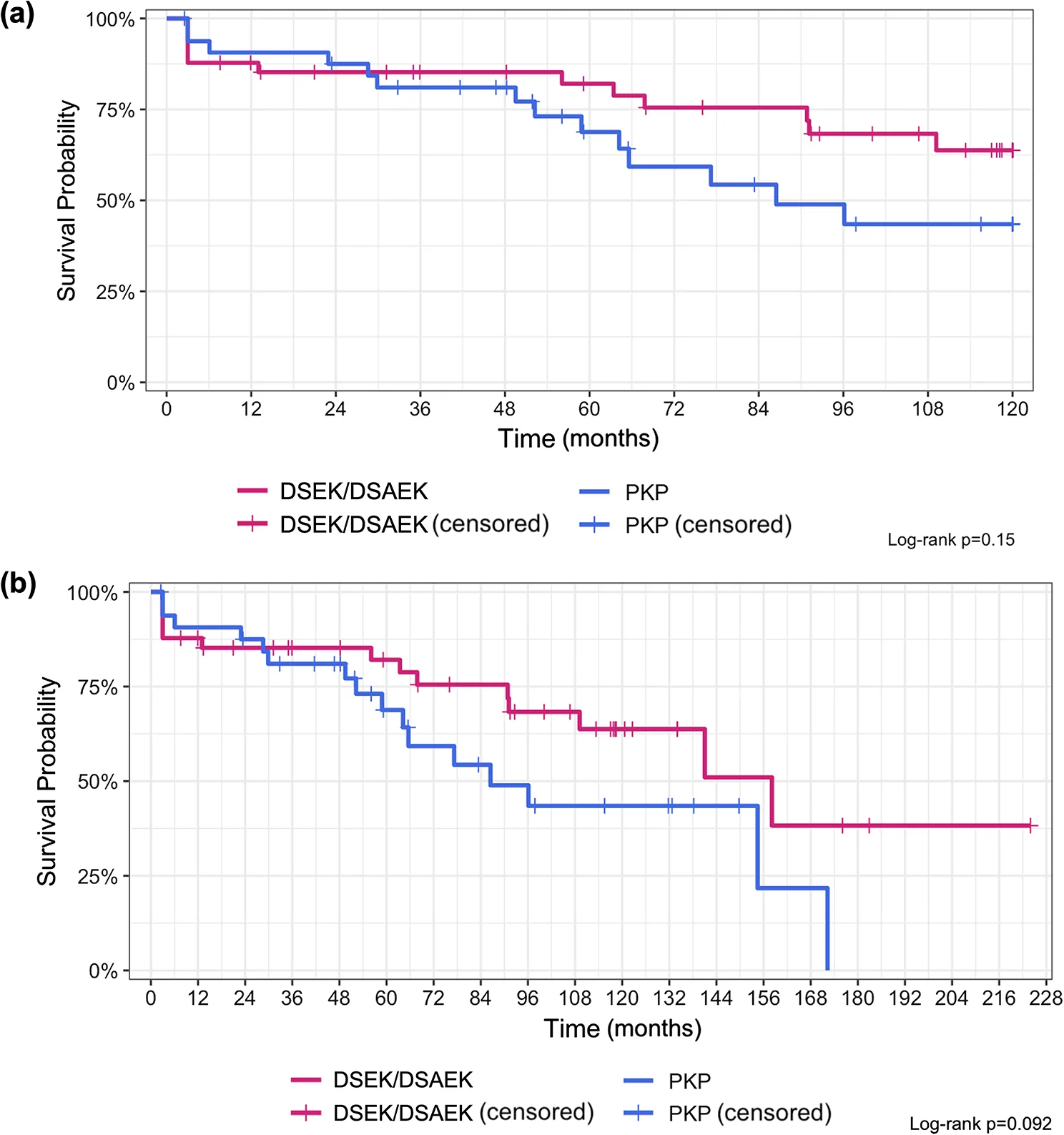
Survival curves for DSEK/DSAEK and PKP. Comparison of Kaplan-Meier survival curves for DSEK/DSAEK and PKP (**a**) at 10 years and (**b**) up to the latest follow-up. DSEK/DSAEK: Descemet stripping (automated) endothelial keratoplasty; PKP: penetrating keratoplasty

**Fig. 3:**
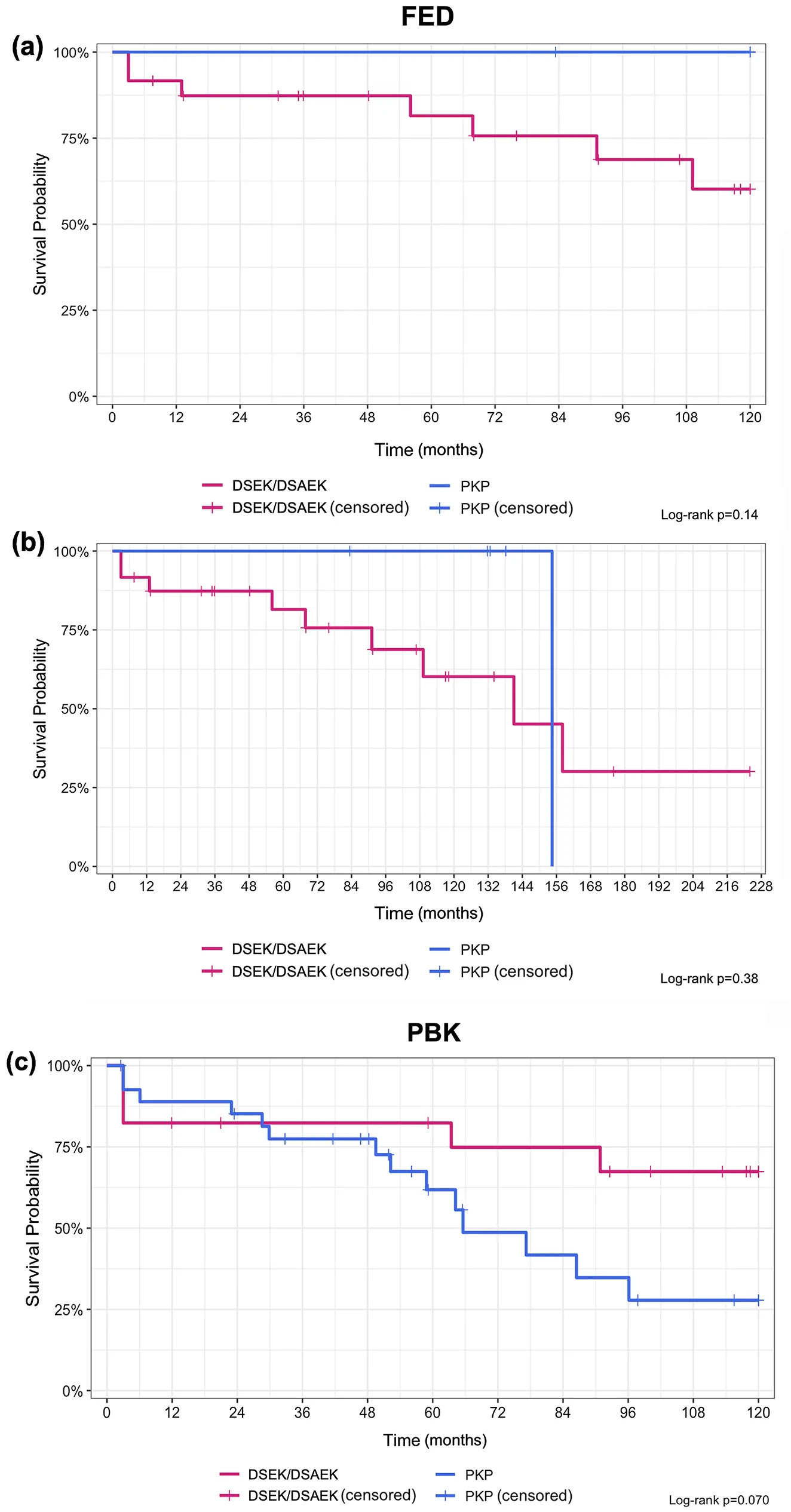

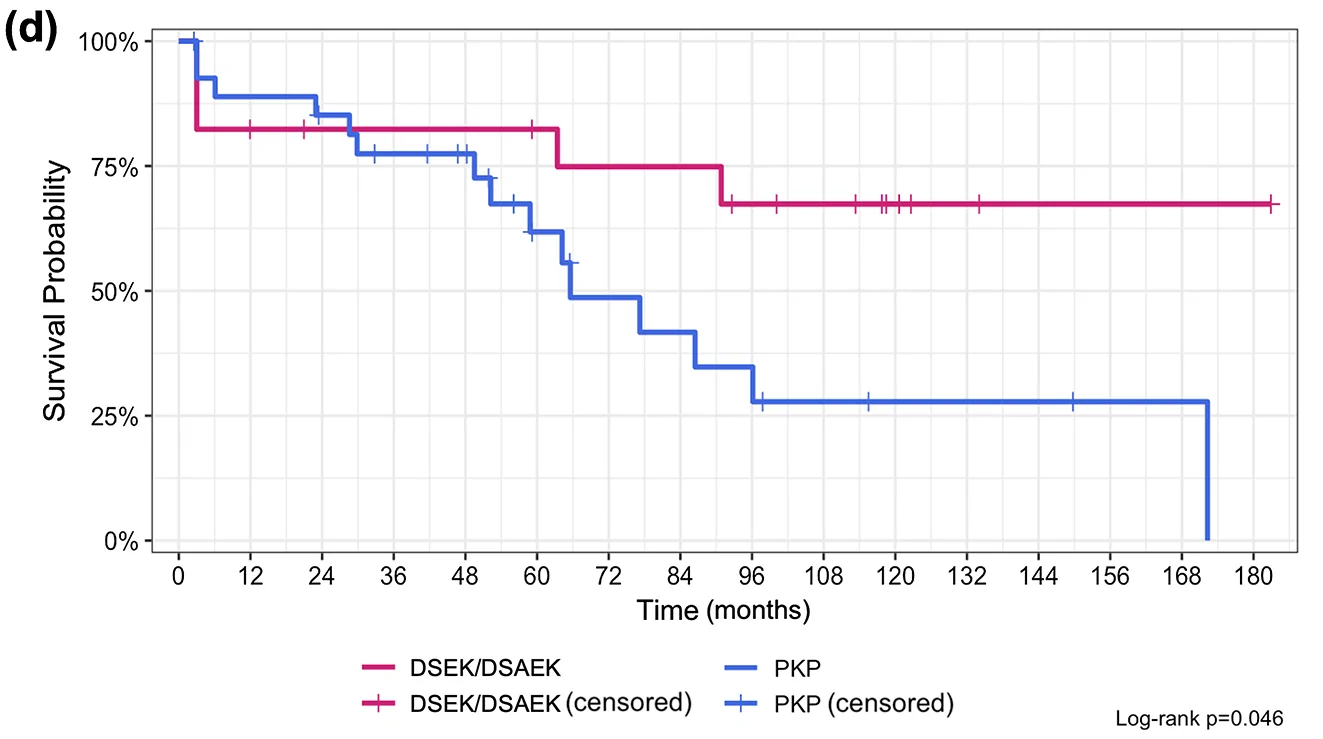
Subgroup analysis of survival curves for DSEK/DSAEK and PKP by diagnosis. Comparison of Kaplan-Meier survival curves for DSEK/DSAEK and PKP in the FED subgroup (**a**) at 10 years and (**b**) up to the latest follow-up, and in the PBK subgroup (**c**) at 10 years and (**d**) up to the latest follow-up. DSEK/DSAEK: Descemet stripping (automated) endothelial keratoplasty; FED: Fuchs endothelial dystrophy; PBK: pseudophakic bullous keratopathy PKP: penetrating keratoplasty

Overall graft survival rates were 76.1% at 5 years, 55.0% at 10 years and 24.1% cumulatively up to the latest follow-up (Figs. 1 and 2; Table 2). DSEK/DSAEK demonstrated higher survival rates at all timepoints, with 82.1% versus 68.8% survival at 5 years (*P* = 0.31), 63.8% versus 43.5% at 10 years (*P* = 0.15) and 38.3% versus 0.0% at the latest follow-up (*P* = 0.092) (Figs. 1 and 2; Table 2). In exploratory subgroup analysis stratified by the diagnosis of FED or PBK, there were no significant differences in 5-year and 10-year survivals between DSEK/DSAEK and PKP (Table 2; Fig. 3). Cumulative graft survival after DSEK/DSAEK was significantly superior to PKP in the PBK subgroup (67.4%, 95% confidence interval [CI]: 47.2–96.1%, vs. 0.0%, 95% CI: 0.0-43.2%, *P* = 0.046) but not the FED subgroup (30.1%, 95% CI: 10.4–87.0%, vs. 0.0%, 95% CI: 0.0-97.5%, *P* = 0.38) (Table 2; Fig. 3), although interpretation is limited by the small subgroup sample sizes. Primary graft failure occurred in 12.2% of DSEK/DSAEK and 6.1% of PKP patients (*P* = 0.37). 14.6% of DSEK/DSAEK and 12.1% of PKP required further transplantation after 10 years (*P* = 0.75).

**Table 2:** Graft survival by type of keratoplasty and diagnosis

Diagnosis	Type of keratoplasty	5-year graft survival (95% CI)	10-year graft survival (95% CI)	Cumulative graft survival (95% CI)
All (*n* = 74)	**All** (*n* = 74)	76.1% (66.4–87.2%)	55.0% (43.0-70.3%)	24.1% (10.0-57.8%)
	**DSEK/DSAEK** (*n* = 41)	82.1% (70.8–95.2%)	63.8% (48.7–83.5%)	38.3% (17.8–82.2%)
	**PKP** (*n* = 33)	68.8% (53.5–88.4%)	43.5% (26.9–70.3%)	0.0% (0.0-44.1%)
	***P*** **values**	0.31	0.15	0.092
FED (*n* = 29)	**All** (*n* = 29)	85.0% (72.4–99.9%)	68.9% (51.5–92.2%)	27.6% (9.1–83.8%)
	**DSEK/DSAEK** (*n* = 24)	81.5% (66.4–100.0%)	60.2% (40.0-90.5%)	30.1% (10.4–87.0%)
	**PKP** (*n* = 5)	100.0% (47.8–100.0%)	100.0% (39.8–100.0%)	0.0% (0.0-97.5%)
	***P*** **values**	0.32	0.14	0.38
PBK (*n* = 45)	**All** (*n* = 45)	70.2% (57.2–86.2%)	45.6% (31.1–66.9%)	22.8% (5.4–96.1%)
	**DSEK/DSAEK** (*n* = 17)	82.4% (66.1–100.0%)	67.4% (47.2–96.1%)	67.4% (47.2–96.1%)
	**PKP** (*n* = 28)	61.8% (44.5–85.9%)	27.8% (12.6–61.6%)	0.0% (0.0-43.2%)
	***P*** **values**	0.29	0.07	0.046*

CI: confidence interval; DSEK/DSAEK: Descemet stripping (automated) endothelial keratoplasty; FED: Fuchs endothelial dystrophy; PBK: pseudophakic bullous keratopathy; PKP: penetrating keratoplasty

*P < 0.05

We focused on factors associated with graft failure at the 10-year mark and 26 graft failure events were included in the regression model which found that older age (hazard ratio [HR] = 1.06, 95% CI: 1.00-1.11, *P* = 0.046) and poorer preoperative BCVA (HR = 1.79, 95% CI: 1.05–3.04, *P* = 0.032) were significant factors in univariate analysis, but not multivariate analysis (Table 3). Shorter operative time was a statistically significant variable (HR = 0.98, 95% CI: 0.96-1.00, *P* = 0.036) but its clinical significance was minimal and therefore not included in the multivariate analysis.

**Table 3:** Cox regression analysis of factors associated with graft failure at 10 years

Factor	Univariate				Multivariate		
	10-year survival	HR	95% CI	*P* values	HR	95% CI	*P* values
Type of keratoplasty							
DSEK/DSAEK	63.8%	1.00					
PKP	43.5%	1.74	0.80–3.78	0.16			
Sex							
Female	54.4%	1.00					
Male	55.7%	0.91	0.40–2.04	0.81			
Age (years)		1.06	1.00-1.11	0.046*	1.04	0.98–1.10	0.18
Diagnosis							
PBK	45.6%	1.00					
FED	68.9%	0.45	0.19–1.08	0.073			
Preoperative BCVA (logMAR)		1.79	1.05–3.04	0.032*	1.64	0.94–2.86	0.082
Preoperative IOP (mmHg)		1.08	0.93–1.25	0.30			
Preoperative CCT (µm)		0.98	0.94–1.01	0.17			
Comorbidities							
Hypertension							
No	61.4%	1.00					
Yes	54.2%	1.22	0.37–4.08	0.74			
Diabetes							
No	69.2%	1.00					
Yes	40.0%	2.10	0.95–4.63	0.066			
Hyperlipidaemia							
No	46.6%	1.00					
Yes	55.9%	1.04	0.47–2.30	0.92			
Ischaemic heart disease							
No	61.4%	1.00					
Yes	27.7%	1.98	0.83–4.71	0.12			
Autoimmune disease							
No	55.4%	1.00					
Yes	50.0%	1.96	0.59–6.54	0.28			
Glaucoma							
No	58.4%	1.00					
Yes	42.2%	1.28	0.51–3.18	0.60			
Duration from listing to surgery (days)		1.00	1.00–1.00	0.85			
Duration of surgery (minutes)		0.98	0.96-1.00	0.036*			
Combined surgery							
With cataract extraction							
No	46.3%	1.00					
Yes	69.8%	0.41	0.16–1.02	0.054			
With IOL implantation							
No	51.4%	1.00					
Yes	58.8%	0.64	0.29–1.42	0.28			
Intraoperative complications							
No	55.6%	1.00					
Yes	46.7%	0.99	0.29–3.30	0.98			
Postoperative complications							
Graft rejection							
No	58.9%	1.00					
Yes	37.5%	1.52	0.57–4.05	0.40			
Graft ulcer							
No	62.7%	1.00					
Yes	0.0%	2.28	0.95–5.42	0.064			
Acutely raised IOP							
No	47.4%	1.00					
Yes	64.0%	0.54	0.24–1.22	0.14			
Chronically raised IOP							
No	69.7%	1.00					
Yes	44.0%	1.83	0.79–4.20	0.16			
Glaucoma							
No	59.6%	1.00					
Yes	41.8%	1.28	0.54–3.05	0.58			
Retinal or choroidal detachment							
No	57.6%	1.00					
Yes	25.0%	2.15	0.64–7.17	0.21			
Endophthalmitis							
No	56.0%	1.00					
Yes	0.00%	4.71	0.62–36.1	0.14			
Peripheral anterior synechiae							
No	59.3%	1.00					
Yes	39.9%	1.18	0.50–2.83	0.71			
Graft displacement							
No	57.0%	1.00					
Yes	0.00%	1.27	0.51–3.17	0.61			
Wound or suture complications							
No	56.8%	1.00					
Yes	40.0%	1.59	0.48–5.31	0.45			

BCVA: best-correct visual acuity; CCT: central corneal thickness; CI: confidence interval; DSEK/DSAEK: Descemet stripping (automated) endothelial keratoplasty; FED: Fuchs endothelial dystrophy; HR: hazard ratio of graft failure; IOL: intraocular lens; IOP: intraocular pressure; PBK: pseudophakic bullous keratopathy; PKP: penetrating keratoplasty

*P < 0.05

### Graft rejection and other complications

The cumulative graft rejection rates at 10 years were 12.2% for DSEK/DSAEK and 9.1% for PKP (*P* = 0.67, Table 4). 40% of DSEK/DSAEK and 67% of PKP rejections were reversed after medical treatment (Fig. 1).

Graft ulcers occurred less commonly in DSEK/DSAEK at 5 years (0.0% vs. 12.1%, *P* = 0.022) and 10 years (4.9% vs. 21.2%, *P* = 0.033). Acute rise in IOP occurred in 58.5% of DSEK/DSAEK eyes compared to 27.3% of PKP eyes within 2 weeks after operation (*P* = 0.0072). More than 50% of patients had chronically elevated IOP, while less than 20% developed new glaucoma following keratoplasty.

Graft displacement occurred only in DSEK/DSAEK (34.1%, *P* = 0.00019), all within 10 days after operation. Nine of 14 dislocated grafts were reattached successfully after a single re-bubbling, 4 grafts required 2 injections, and the remaining graft required 3 injections. Suture-related complications occurred only in PKP (15.2%, *P* = 0.0098). Graft outcomes and complication profiles from exploratory subgroup analyses stratified by diagnosis are included in Supplementary Tables 1 and Supplementary Table 2.

### Vision-related outcomes

Postoperative observed BCVA was significantly better for the DSEK/DSAEK group at 5 years (logMAR 0.68 ± 0.70 vs. 1.46 ± 1.04, *P* = 0.0060) but this difference diminished by 10 years (Table 4). Subgroup analysis by diagnosis showed that postoperative BCVA was superior after DSEK/DSAEK in the FED subgroup only (logMAR 0.42 ± 0.25 vs. 0.87 ± 0.29, *P* = 0.031) (Supplementary Tables 1 and Supplementary Table 2). DSEK/DSAEK (β = 0.78 for PKP, 95% CI: 0.27–1.30, *P* = 0.0045), the diagnosis of FED (β=-0.85, 95% CI: -1.36 to -0.34, *P* = 0.0022) and better preoperative BCVA (β = 0.72, 95% CI: 0.40–1.05, *P* = 0.000076) were associated with better postoperative BCVA at 5 years in univariate regression analysis (Table 5). DSEK/DSAEK remained significant after adjusting for the preoperative diagnosis but not after adjusting for preoperative BCVA (Table 5). Postoperative IOP and CCT were similar between both groups (both *P* > 0.05).

**Table 4:** Graft outcomes and complications

Outcome	All (*n* = 74)	DSEK/DSAEK (*n* = 41)	PKP (*n* = 33)	*P* values
Primary graft failure	7 (9.5%)	5 (12.2%)	2 (6.1%)	0.37
Re-graft				
5 years	6 (8.1%)	4 (9.8%)	2 (6.1%)	0.56
10 years	10 (13.5%)	6 (14.6%)	4 (12.1%)	0.75
BCVA (logMAR)				
5 years	1.05 ± 0.96	0.68 ± 0.70	1.46 ± 1.04	0.0060*
10 years	1.25 ± 1.18	1.10 ± 1.11	1.51 ± 1.25	0.42
IOP (mmHg)				
5 years	14.1 ± 3.79	13.5 ± 3.83	15.0 ± 3.54	0.22
10 years	13.8 ± 2.85	13.7 ± 2.96	14.1 ± 2.55	0.71
CCT (µm)				
5 years	593 ± 109	604 ± 92.4	572 ± 131	0.52
10 years	590 ± 59.6	608 ± 41.7	518 ± 65.5	0.39
Postoperative complications				
Graft rejection				
5 years	4 (5.4%)	2 (4.9%)	2 (6.1%)	0.82
10 years	8 (10.8%)	5 (12.2%)	3 (9.1%)	0.67
Graft ulcer				
5 years	4 (5.4%)	0 (0.0%)	4 (12.1%)	0.022*
10 years	9 (12.2%)	2 (4.9%)	7 (21.2%)	0.033*
Acutely raised IOP	33 (44.6%)	24 (58.5%)	9 (27.3%)	0.0072*
Chronically raised IOP				
5 years	41 (55.4%)	21 (51.2%)	20 (60.6%)	0.42
10 years	41 (55.4%)	21 (51.2%)	20 (60.6%)	0.42
Glaucoma				
5 years	12 (16.2%)	8 (19.5%)	4 (12.1%)	0.39
10 years	14 (18.9%)	10 (24.4%)	4 (12.1%)	0.18
Retinal or choroidal detachment				
5 years	3 (4.1%)	1 (2.4%)	2 (6.1%)	0.43
10 years	4 (5.4%)	2 (4.9%)	2 (6.1%)	0.82
Endophthalmitis				
5 years	1 (1.4%)	0 (0.0%)	1 (3.0%)	0.26
10 years	1 (1.4%)	0 (0.0%)	1 (3.0%)	0.26
Peripheral anterior synechiae				
5 years	16 (21.6%)	12 (29.3%)	4 (12.1%)	0.075
10 years	16 (21.6%)	12 (29.3%)	4 (12.1%)	0.075
Graft displacement				
5 years	14 (18.9%)	14 (34.1%)	0 (0.0%)	0.00019*
10 years	14 (18.9%)	14 (34.1%)	0 (0.0%)	0.00019*
Suture-related complications				
5 years	5 (6.8%)	0 (0.0%)	5 (15.2%)	0.0098*
10 years	5 (6.8%)	0 (0.0%)	5 (15.2%)	0.0098*

BCVA: best-correct visual acuity; CCT: central corneal thickness; DSEK/DSAEK: Descemet stripping (automated) endothelial keratoplasty; IOP: intraocular pressure; PKP: penetrating keratoplasty

*P < 0.05

**Table 5:** Linear regression analysis of factors associated with postoperative BCVA (logMAR) at 5 years

Variable	Univariate			Multivariate (Model 1)			Multivariate (Model 2)		
	β	95% CI	*P* values	β	95% CI	*P* values	β	95% CI	*P* values
Type of keratoplasty									
DSEK/DSAEK	Ref			Ref			Ref		
PKP	0.78	0.27–1.30	0.0045*	0.56	0.047–1.08	0.038*	0.23	-0.29 to 0.76	0.38
Diagnosis									
PBK	Ref			Ref			Ref		
FED	-0.85	-1.36 to -0.34	0.0022*	-0.66	-1.18 to -0.13	0.019*	-0.54	-1.03 to -0.058	0.034*
Preoperative BCVA (logMAR)	0.72	0.40–1.05	0.000076*				0.55	0.19–0.90	0.0042*

BCVA: best-correct visual acuity; CI: confidence interval; DSEK/DSAEK: Descemet stripping (automated) endothelial keratoplasty; FED: Fuchs endothelial dystrophy; PBK: pseudophakic bullous keratopathy; PKP: penetrating keratoplasty; Ref: reference

*P < 0.05

## Discussion

Our study presents real-world surgical outcomes with relatively long follow-up duration and data originating from an Asian cohort that remains underrepresented in the literature. We showed that both primary DSEK/DSAEK and PKP provided meaningful long-term graft survival among this cohort of FED or PBK patients. Our 5-year survival rates of 82.1% for DSEK/DSAEK and 68.8% for PKP were higher than other reported Asian cohorts. The Singaporean cohorts demonstrated better survival for DSAEK (78.4–79.4%) compared to PKP (54.6–66.5%) at 5 years [10, 11], while the Malaysian cohort revealed no significant difference over a period of more than 5 years (DSEK 73.1% and PKP 53.1%) [12]. However, our survival figures were not as high as the Dutch National Registry Study (DSEK/DSAEK 93.4% and PKP 89.7%) [13], which may be explained by shorter globes with shallower anterior chambers in Asian eyes posing more difficulty for DSEK/DSAEK [2]. Our results were consistent with a recent systematic review that concluded a trend towards better 5-year survival for DSEK/DSAEK [5].

Long-term data comparing DSEK/DSAEK and PKP is lacking. Our study provided head-to-head comparison data from a single-centre Asian cohort with contemporaneous recruitment and one of the longest follow-up periods, showing 10-year survivals of 63.8% for DSEK/DSAEK and 43.5% for PKP, which were similar to previous non-comparative cohorts for DSEK/DSAEK (62–92%) and PKP (20–90%) [5]. The Australian Corneal Graft Registry showed 10-year survivals of 36% for DSEK/DSAEK and 59% for PKP and there was no reported survival of DSEK/DSAEK beyond around 14 years [14]. Their inferior DSEK/DSAEK survival was partially attributed to the inclusion of the earliest DSEK patients in 2006 which had poorer outcomes. Our centre similarly started performing DSEK in 2006 and our longest surviving DSEK has been functioning for over 18 years, showing promising extra long-term performance. The German cohort reported 10-year survivals of 73% for DSAEK and 92% for PKP among FED patients; however, direct comparison is difficult because their group defined graft failure as a loss in function requiring a re-graft, which would inevitably overestimate survival due to reluctance in re-graft for various reasons [15]. To the best of our knowledge, no other studies have directly compared survival of primary DSEK/DSAEK versus PKP at 10 years or beyond.

There was a trend towards poorer DSEK/DSAEK survival in the early postoperative months with survival curves of DSEK/DSAEK and PKP crossing over before 30 months. Poorer 2-year survival was similarly demonstrated in the Dutch and British cohorts [13, 16]. Our survival curve crossover time was slightly later than cohorts from Malaysia (around 15 months) and Taiwan (around 500 days) [12, 17]. This may be partially explained by higher rates of primary failure in DSEK/DSAEK (12.2% vs. 6.1%) and early DSEK/DSAEK complications [18], including acute IOP rise due to pupillary block confirmed clinically (58.5%) and graft displacement (34.1%), as opposed to an overall postoperative complication rate of 11.5% in Malaysia and graft dislocation rate of 28.0% in Taiwan [12, 17]. However, these factors were not associated with graft failure in our analysis, indicating alternative factors.

Older age was associated with increased risk of graft failure. There has been mixed evidence on the effect of age depending on the inclusion of paediatric grafts, with some studies suggesting poorer outcomes in the elderly, potentially due to increased risks of infection and trauma, lower host endothelial cell density (ECD), drug non-compliance and systemic comorbidities [19–22]. On the other hand, other studies have shown that younger age may predict failure due to higher rejection rates and more complicated anatomy in paediatric grafts [23, 24].

DSEK/DSAEK achieved a better postoperative BCVA at 5 years, which is consistent with findings from Malaysia at 2 years [12], Singapore and Japan at 5 years [11, 25], and the Netherlands at 3 years for FED and 5 years for PBK [13], as well as a meta-analysis of comparative studies with follow-up periods up to 5 years [4]. In our cohort, the type of keratoplasty was associated with 5-year postoperative BCVA in univariate analysis and after adjusting for diagnosis, however, this difference attenuated after adjusting for preoperative BCVA. This may reflect faster visual recovery after DSEK/DSAEK as well as a trend towards earlier intervention with DSEK/DSAEK, as evidenced by a poorer preoperative BCVA in PKP patients who are likely to have more advanced disease with anterior corneal involvement precluding DSEK/DSAEK. All patients in the DSEK/DSAEK group with FED were phakic so combined cataract surgery was performed for most of them, which may also confound the better postoperative BCVA in this group at 5 years. However, we are the first to report that this superior effect diminished at 10 years with both DSEK/DSAEK and PKP patients seeing equally well.

PKP eyes were more susceptible to ulcers. The higher infection rate in PKP may be attributable to more suture-related complications, greater use of topical steroids, chronic application of topical anti-glaucomatous medications, poorer ocular surface, as well as more neurotrophic keratitis due to decreased nerve fibre density after regeneration [11, 26, 27].

Chronically raised IOP was a common burden for keratoplasty patients, affecting 51.2% of DSEK/DSAEK and 60.6% of PKP, which were higher than reported rates of 38.0% for DSEK/DSAEK and 53.4% for PKP [28]. This was likely due to a significant portion of eyes with pre-existing glaucoma and a more stringent IOP criteria of 21 mmHg. The 5-year glaucoma rate for DSEK/DSAEK (19.5%) was higher than PKP (12.1%), but the rates were comparable to results from Singapore (15.8% for DSAEK and 15.3% for PKP) [10]. Although new development of glaucoma was documented in only 18.9% of patients, this was likely an underestimation as patients with poor vision and fixation precluded reliable assessment with optical coherence tomography.

There were a number of limitations for our study. Firstly, our study was limited by a small sample size as donor grafts were limited. As one of the earliest centres to perform DSEK/DSAEK in Hong Kong, we did not receive a priority in receiving corneal tissues, nor were we allowed to import foreign corneas for transplantation. This significantly reduced our ability to increase the sample size. As a result, the study may be underpowered to detect moderate survival differences between DSEK/DSAEK and PKP. Secondly, as a retrospective cohort study, there were naturally more FED patients who received DSEK/DSAEK than PKP, and DSEK/DSAEK patients had better preoperative BCVA, which were consistent with observations from other reported cohorts [11, 13, 29]. DSEK/DSAEK patients were also more likely to have combined cataract surgery. These baseline differences limit direct comparison and causal interpretation. Nonetheless, neither the preoperative diagnosis nor the type of keratoplasty was associated with graft failure in our regression analyses. Subgroup analyses stratified by diagnosis and adjusted regression analysis were also used to address these potential confounders. These subgroup analyses were exploratory and limited by small sample sizes, especially in the FED subgroup where only 5 eyes received PKP. Thirdly, most EK grafts in our cohort were manually dissected, as the Hospital Authority Eye Bank began pre-cutting grafts with a microkeratome only in 2008. With the increasing adoption of DSAEK and, more recently, Descemet membrane endothelial keratoplasty (DMEK), our long-term results on DSEK/DSAEK may not be entirely representative. However, they still serve as a valuable reference for the long-term outcomes of a more established technique. Pre-stripped DMEK grafts have only become available in Hong Kong since 2021, and due to the low donation rate, surgeons have been cautious about the risk of graft loss during preparation. Consequently, there is currently no long-term data available, and DSEK/DSAEK continues to be the most commonly performed EK procedure in Hong Kong. Finally, future studies can also explore the effect of donor factors such as donor age, sex, ECD, graft size, death-to-preservation time, preservation-to-use time and comorbidities [30, 31]. In this retrospective study, most donor factors were not available because they were centrally stored by the Hospital Authority Eye Bank.

## Conclusions

Both primary DSEK/DSAEK and PKP provided meaningful long-term graft survival in this Hong Kong cohort of patients with FED or PBK. DSEK/DSAEK eyes had better observed BCVA at 5 years, although this association was attenuated after adjustment for preoperative BCVA, and the visual difference was no longer apparent at 10 years. DSEK/DSAEK was associated with fewer graft ulcers, while rejection and re-graft rates were similar between groups. These findings should be interpreted in the context of the retrospective design, baseline differences between treatment groups, small subgroup sample sizes, and the historical DSEK/DSAEK techniques represented in this cohort.

## Supplementary Information

Below is the link to the electronic supplementary material.

Supplementary Material 1

## Data Availability

The datasets used and/or analysed during the current study are available from the corresponding author on reasonable request.
