# Supplementary material for: Ten-year outcomes of endothelial keratoplasty versus penetrating keratoplasty in Hong Kong: a retrospective cohort study
Source: BMC Ophthalmol. 2026 Sep 11;26:566. doi: 10.1186/s12886-026-05332-4 (PMC13573452; doi:10.1186/s12886-026-05332-4)
Supplement: Supplementary file 1 [file 12886_2026_5332_MOESM1_ESM.docx]

**Supplementary Materials**

**Supplementary Table 1** Graft outcomes and complications in the FED subgroup

| Outcome | All  (n=29) | DSEK/DSAEK  (n=24) | PKP  (n=5) | *P* values |
| --- | --- | --- | --- | --- |
| Primary graft failure | 2 (6.9%) | 2 (8.3%) | 0 (0.0%) | 0.50 |
| Re-graft |  |  |  |  |
| 5 years | 3 (10.3%) | 3 (12.5%) | 0 (0.0%) | 0.40 |
| 10 years | 5 (17.2%) | 5 (20.8%) | 0 (0.0%) | 0.26 |
| BCVA (logMAR) |  |  |  |  |
| 5 years | 0.54 ± 0.32 | 0.42 ± 0.25 | 0.87 ± 0.29 | 0.031* |
| 10 years | 0.79 ± 0.54 | 0.76 ± 0.59 | 0.86 ± 0.39 | 0.75 |
| IOP (mmHg) |  |  |  |  |
| 5 years | 14.3 ± 3.60 | 14.6 ± 3.71 | 13.3 ± 2.95 | 0.51 |
| 10 years | 13.9 ± 3.15 | 14.2 ± 3.37 | 13.4 ± 2.48 | 0.68 |
| Postoperative complications |  |  |  |  |
| Graft rejection |  |  |  |  |
| 5 years | 2 (6.9%) | 1 (4.2%) | 1 (20.0%) | 0.20 |
| 10 years | 6 (20.7%) | 4 (16.7%) | 2 (40.0%) | 0.24 |
| Graft ulcer |  |  |  |  |
| 5 years | 0 (0.0%) | 0 (0.0%) | 0 (0.0%) | NA |
| 10 years | 1 (3.4%) | 1 (4.2%) | 0 (0.0%) | 0.64 |
| Acutely raised IOP | 18 (62.1%) | 16 (66.7%) | 2 (40.0%) | 0.26 |
| Chronically raised IOP |  |  |  |  |
| 5 years | 12 (41.4%) | 10 (41.7%) | 2 (40.0%) | 0.95 |
| 10 years | 12 (41.4%) | 10 (41.7%) | 2 (40.0%) | 0.95 |
| Glaucoma |  |  |  |  |
| 5 years | 4 (13.8%) | 4 (16.7%) | 0 (0.0%) | 0.33 |
| 10 years | 6 (20.7%) | 6 (25.0%) | 0 (0.0%) | 0.21 |
| Retinal or choroidal detachment | | | | |
| 5 years | 0 (0.0%) | 0 (0.0%) | 0 (0.0%) | NA |
| 10 years | 1 (3.4%) | 1 (4.2%) | 0 (0.0%) | 0.64 |
| Endophthalmitis |  |  |  |  |
| 5 years | 0 (0.0%) | 0 (0.0%) | 0 (0.0%) | NA |
| 10 years | 0 (0.0%) | 0 (0.0%) | 0 (0.0%) | NA |
| Peripheral anterior synechiae |  |  |  |  |
| 5 years | 8 (27.6%) | 8 (33.3%) | 0 (0.0%) | 0.13 |
| 10 years | 8 (27.6%) | 8 (33.3%) | 0 (0.0%) | 0.13 |
| Graft displacement |  |  |  |  |
| 5 years | 10 (34.5%) | 10 (41.7%) | 0 (0.0%) | 0.075 |
| 10 years | 10 (34.5%) | 10 (41.7%) | 0 (0.0%) | 0.075 |
| Suture-related complications |  |  |  |  |
| 5 years | 1 (3.4%) | 0 (0.0%) | 1 (20.0%) | 0.026* |
| 10 years | 1 (3.4%) | 0 (0.0%) | 1 (20.0%) | 0.026* |

BCVA: best-correct visual acuity; FED: Fuchs endothelial dystrophy; DSEK/DSAEK: Descemet stripping (automated) endothelial keratoplasty; IOP: intraocular pressure; PKP: penetrating keratoplasty

**P*<0.05

**Supplementary Table 2** Graft outcomes and complications in the PBK subgroup

| Outcome | All  (n=45) | DSEK/DSAEK  (n=17) | PKP  (n=28) | *P* values |
| --- | --- | --- | --- | --- |
| Primary graft failure | 5 (11.1%) | 3 (17.6%) | 2 (7.1%) | 0.28 |
| Re-graft |  |  |  |  |
| 5 years | 3 (6.7%) | 1 (5.9%) | 2 (7.1%) | 0.87 |
| 10 years | 5 (11.1%) | 1 (5.9%) | 4 (14.3%) | 0.38 |
| BCVA (logMAR) |  |  |  |  |
| 5 years | 1.39 ± 1.09 | 1.01 ± 0.93 | 1.64 ± 1.11 | 0.13 |
| 10 years | 1.68 ± 1.43 | 1.48 ± 1.40 | 1.95 ± 1.42 | 0.58 |
| IOP (mmHg) |  |  |  |  |
| 5 years | 13.9 ± 3.93 | 11.9 ± 3.38 | 15.6 ± 3.53 | 0.026* |
| 10 years | 13.7 ± 2.40 | 13.0 ± 2.15 | 15.2 ± 2.25 | 0.31 |
| Postoperative complications |  |  |  |  |
| Graft rejection |  |  |  |  |
| 5 years | 2 (4.4%) | 1 (5.9%) | 1 (3.6%) | 0.72 |
| 10 years | 2 (4.4%) | 1 (5.9%) | 1 (3.6%) | 0.72 |
| Graft ulcer |  |  |  |  |
| 5 years | 4 (8.9%) | 0 (0.0%) | 4 (14.3%) | 0.10 |
| 10 years | 8 (17.8%) | 1 (5.9%) | 7 (25.0%) | 0.10 |
| Acutely raised IOP | 15 (33.3%) | 8 (47.1%) | 7 (25.0%) | 0.13 |
| Chronically raised IOP |  |  |  |  |
| 5 years | 29 (64.4%) | 11 (64.7%) | 18 (64.3%) | 0.98 |
| 10 years | 29 (64.4%) | 11 (64.7%) | 18 (64.3%) | 0.98 |
| Glaucoma |  |  |  |  |
| 5 years | 8 (17.8%) | 4 (23.5%) | 4 (14.3%) | 0.43 |
| 10 years | 8 (17.8%) | 4 (23.5%) | 4 (14.3%) | 0.43 |
| Retinal or choroidal detachment | | | | |
| 5 years | 3 (6.7%) | 1 (5.9%) | 2 (7.1%) | 0.87 |
| 10 years | 3 (6.7%) | 1 (5.9%) | 2 (7.1%) | 0.87 |
| Endophthalmitis |  |  |  |  |
| 5 years | 1 (2.2%) | 0 (0.0%) | 1 (3.6%) | 0.43 |
| 10 years | 1 (2.2%) | 0 (0.0%) | 1 (3.6%) | 0.43 |
| Peripheral anterior synechiae |  |  |  |  |
| 5 years | 8 (17.8%) | 4 (23.5%) | 4 (14.3%) | 0.43 |
| 10 years | 8 (17.8%) | 4 (23.5%) | 4 (14.3%) | 0.43 |
| Graft displacement |  |  |  |  |
| 5 years | 4 (8.9%) | 4 (23.5%) | 0 (0.0%) | 0.0072* |
| 10 years | 4 (8.9%) | 4 (23.5%) | 0 (0.0%) | 0.0072* |
| Suture-related complications |  |  |  |  |
| 5 years | 4 (8.9%) | 0 (0.0%) | 4 (14.3%) | 0.10 |
| 10 years | 4 (8.9%) | 0 (0.0%) | 4 (14.3%) | 0.10 |

BCVA: best-correct visual acuity; DSEK/DSAEK: Descemet stripping (automated) endothelial keratoplasty; IOP: intraocular pressure; PBK: pseudophakic bullous keratopathy; PKP: penetrating keratoplasty

**P*<0.05
